# Lithium–Metal Batteries Using Sustainable Electrolyte Media and Various Cathode Chemistries

**DOI:** 10.1021/acs.energyfuels.1c00927

**Published:** 2021-05-20

**Authors:** Vittorio Marangon, Luca Minnetti, Matteo Adami, Alberto Barlini, Jusef Hassoun

**Affiliations:** † University of Ferrara, Department of Chemical and Pharmaceutical Sciences, Via Fossato di Mortara 17, 44121 Ferrara, Italy; ‡ Graphene Laboratories, Istituto Italiano di Tecnologia, Via Morego 30, 16163 Genova, Italy

## Abstract

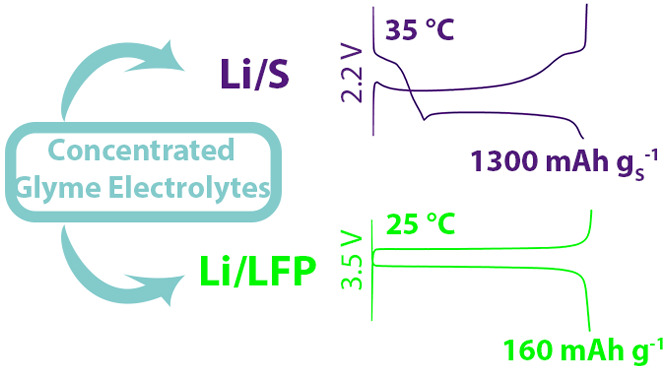

Lithium–metal batteries employing concentrated glyme-based electrolytes and two different cathode chemistries are herein evaluated in view of a safe use of the highly energetic alkali-metal anode. Indeed, diethylene-glycol dimethyl-ether (DEGDME) and triethylene-glycol dimethyl-ether (TREGDME) dissolving lithium bis(trifluoromethanesulfonyl)imide (LiTFSI) and lithium nitrate (LiNO_3_) in concentration approaching the solvents saturation limit are used in lithium batteries employing either a conversion sulfur–tin composite (S:Sn 80:20 w/w) or a Li^+^ (de)insertion LiFePO_4_ cathode. Cyclic voltammetry (CV) and electrochemical impedance spectroscopy (EIS) clearly show the suitability of the concentrated electrolytes in terms of process reversibility and low interphase resistance, particularly upon a favorable activation. Galvanostatic measurements performed on lithium–sulfur (Li/S) batteries reveal promising capacities at room temperature (25 °C) and a value as high as 1300 mAh g_S_^–1^ for the cell exploiting the DEGDME-based electrolyte at 35 °C. On the other hand, the lithium–LiFePO_4_ (Li/LFP) cells exhibit satisfactory cycling behavior, in particular when employing an additional reduction step at low voltage cutoff (i.e., 1.2 V) during the first discharge to consolidate the solid electrolyte interphase (SEI). This procedure allows a Coulombic efficiency near 100%, a capacity approaching 160 mAh g^–1^, and relevant retention particularly for the cell using the TREGDME-based electrolyte. Therefore, this work suggests the use of concentrated glyme-based electrolytes, the fine-tuning of the operative conditions, and the careful selection of active materials chemistry as significant steps to achieve practical and safe lithium–metal batteries.

## Introduction

Li-ion batteries power a wide array of electronic devices, from portable systems such as laptops and smartphones to hybrid (HEVs) and fully electric vehicles (EVs).^1,2^ The research on Li-ion batteries has led to the achievement of a remarkable energy density, i.e., 260 Wh kg^–1^, and a long cycle life.^3,4^ However, an increasing demand for energy with the purpose of extending the driving range of EVs has renewed interest in the metallic lithium, which offers a high theoretical capacity (3860 mAh g^–1^) and the lowest redox potential (−3.04 V vs SHE) among the various electrodes proposed as the battery anode.^5^ Despite the various advantages, the application of lithium in a rechargeable battery has so far been hindered by the formation of dendritic structures due to heterogeneous deposition of lithium at the metal surface during charge that can lead to short circuits and hazards.^6^ The most relevant solutions proposed to overcome this challenging issue and ensure efficient and safe discharge–charge cycling of the battery are represented by the addition to the electrolyte of sacrificial agents such as lithium nitrate (LiNO_3_), that can be reduced at the lithium surface to protect the metallic anode by the formation of a suitable solid electrolyte interphase (SEI) film.^7−9^ A further relevant breakthrough was achieved by the replacement of carbonate-based solvents with more stable and less volatile poly(ethylene oxide)s or end-capped glymes (CH_3_O(CH_2_CH_2_O)_*n*_CH_3_).^10−15^ Remarkable improvement of the safety content of the cell was furthermore obtained by increasing the salt concentration, in particular using the glyme-based electrolytes, in order to decrease the flammability and the volatility, holding at the same time long cycle life and high Coulombic efficiency.^16−19^ In this regard, in our previous study we have characterized the chemical and electrochemical properties of highly concentrated di- and triglyme-based electrolytes employing the conductive salt lithium bis(trifluoromethanesulfonyl)imide (LiTFSI) and LiNO_3_ in concentrations approaching the solvent saturation limit.^20^ The study focused on the performance of the new electrolyte media in a Li/O_2_ battery, particularly in terms of electrode/electrolyte interphase effects on the cycling behavior of the cell. The data of the above research principally suggested the triglyme-based electrolyte as a promising candidate for application in Li/O_2_ cells due to its unique properties which include a remarkably low volatility and enhanced interphase stability,^20^ thus in agreement with other literature papers.^21,22^ Indeed, according to X-ray photoelectron spectroscopy (XPS) and electrochemical impedance spectroscopy (EIS), the enhanced characteristics of the triglyme-based electrolyte compared to the diglyme-based one in a Li/O_2_ cell have been principally attributed to the formation of a stable SEI, in particular on the Li metal.^20^

Herein, we originally extend the investigation of these highly concentrated electrolyte media to different cathode chemistries which can be employed in new configurations of lithium–metal batteries, that is, the ones using the conversion electrochemical process related to sulfur^23,24^ and the Li^+^ (de)insertion mechanism associated with a LiFePO_4_ olivine cathode.^3,25^ Therefore, the present study focuses on the electrochemical performances of the new electrolytes in advanced lithium cells using the high-performance sulfur composite with low amount of electrochemically inactive, conductive tin metal (S:Sn 80:20 w/w)^26^ and the advanced carbon-coated LiFePO_4_ cathode.^27^ The results of the study may actually shed light on possible applications of the highly concentrated glyme-based electrolytes for achieving new rechargeable batteries with remarkable safety content using the highly energetic, yet challenging, lithium–metal anode.

## Experimental Section

### Materials

Lithium bis(trifluoromethanesulfonyl)imide (LiTFSI, Sigma-Aldrich) and lithium nitrate (LiNO_3_, Sigma-Aldrich) salts were dissolved in diethylene-glycol dimethyl-ether (DEGDME, CH_3_O(CH_2_CH_2_O)_2_CH_3_, Sigma-Aldrich) and triethylene-glycol dimethyl-ether (TREGDME, CH_3_O(CH_2_CH_2_O)_3_CH_3_, Sigma-Aldrich) solvents at room temperature overnight under magnetic stirring inside an Ar-filled glovebox (MBraun, O_2_ and H_2_O content below 1 ppm). The final concentration of each salt was 1.5 mol kg_solvent_^–1^ in DEGDME and 2 mol kg_solvent_^–1^ in TREGDME, that is, amounts approaching the saturation limit of the solvents. Prior to use, LiTFSI and LiNO_3_ were dried at 110 °C for 24 h under vacuum, while DEGDME and TREGDME solvents were dried under molecular sieves (3 Å, rods, size 1/16 in., Honeywell Fluka) until a water content lower than 10 ppm was achieved as measured by a 899 Karl Fischer coulometer (Metrohm). The highly concentrated electrolyte solutions are subsequently indicated as DEGDME_HCE and TREGDME_HCE. The analyses of the chemical and electrochemical properties of the electrolytes are reported in a previous work.^20^

The synthesis of the sulfur composite (S:Sn 80:20) was achieved in a previous work through a physical mixing and melting process of elemental sulfur (80% wt, ≥99.5%, Riedel-de Haën) and nanometric tin powder (20% wt, <150 nm, ≥99% trace metal basis, Sigma-Aldrich) at 120 °C,^26^ while the LiFePO_4_ (LFP) material was developed by advanced lithium electrochemistry (Aleees Taiwan, model A1100) and characterized by a carbon content of about 5%.^27^

### Electrochemical Measurements

The electrochemical tests were carried out in CR2032 coin-type cells assembled in an Ar-filled glovebox (MBraun, O_2_ and H_2_O content below 1 ppm) by employing a 14 mm diameter lithium disk as the anode. The S:Sn 80:20 and LFP electrodes were obtained by NMP-solvent casting of the active materials (80% wt), Super P carbon (10% wt, Timcal) and polyvinilidene fluoride (10% wt, Solef^Ⓡ^ 6020 PVDF) on a porous carbon-cloth foil (GDL, ELAT 1400, MTI Corp.) or an aluminum current collector, respectively. The active material loading was of about 1.3 mg cm^−2^ for S:Sn 80:20 and 4.5 mg cm^−2^ for LFP as normalized to the electrode geometric area (1.54 cm^2^). The cathodes were separated from the lithium anode by a 16 mm Celgard (2400) foil soaked with the electrolyte solution (either DEGDME_HCE or TREGDME_HCE, see below for the related amounts) in the Li/S:Sn 80:20 cells, while they were separated by two GF/A glass fiber Whatman 16 mm disks soaked with the electrolyte solution (either DEGDME_HCE or TREGDME_HCE) in the Li/LFP cells.

Cyclic voltammetry (CV) was performed at a scan rate of 0.1 mV s^–1^ in the 1.8–2.8 V vs Li^+^/Li potential range for the S:Sn 80:20 electrode and in the 2.7–3.9 V vs Li^+^/Li potential range for the LFP one. Electrochemical impedance spectra were collected at the open circuit voltage (OCV) condition of the cell, as well as after 1, 5, and 10 CV cycles, and were analyzed through the nonlinear least-squares (NLLS) fitting method via the Boukamp software (χ^2^ values of the order of 10^–4^ or lower).^28,29^ EIS was performed by applying to the cells an alternate voltage signal with an amplitude of 10 mV within the frequency range from 500 kHz to 100 mHz. All of the CV and EIS measurements were performed by using a VersaSTAT MC Princeton Applied Research (PAR, AMETEK) instrument.

The Li/S:Sn 80:20 cells were tested through galvanostatic cycling measurements carried out at the constant current rate of C/5 at 25 and 35 °C and of 1C at 35 °C (1C = 1675 mA g_S_^–1^). The cells cycled at the current of C/5 employed 60 μL of electrolyte solution and a voltage range of 1.9–2.8 V, while voltage limits of 1.6 and 2.8 V and an electrolyte/sulfur ratio of 20 μL mg^–1^ were adopted for the cells tested at 1C. The galvanostatic cycling measurements of the Li/LFP cells were performed at the constant current rate of C/5 (1C = 170 mA g^–1^) at room temperature (25 °C), in a 2.7–3.9 V voltage range. Additional tests were carried out at C/5 and C/3 by exploiting a voltage range between 1.2 and 3.9 V during the first charge–discharge cycle and between 2.7 and 3.9 V for the subsequent ones. All of the galvanostatic cycling measurements were performed by using a MACCOR series 4000 battery test system.

## Results and Discussion

**Figure 1:**
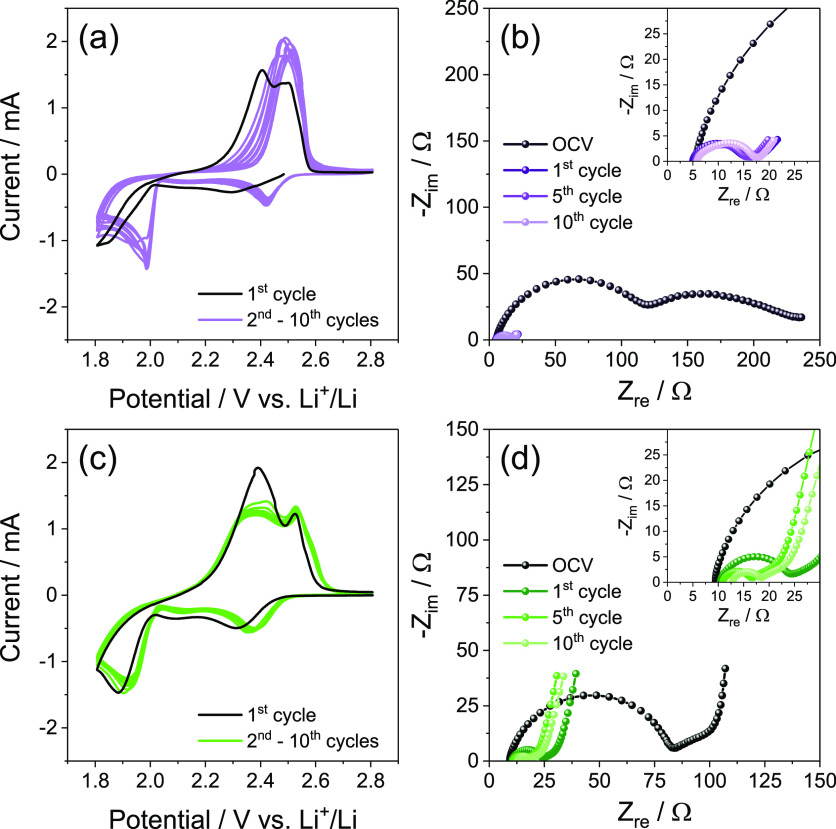
(a, c) Cyclic voltammetry (CV) and (b, d) electrochemical impedance spectroscopy (EIS) measurements performed on Li/electrolyte/S:Sn 80:20 cells employing either (a, b) DEGDME_HCE or (c, d) TREGDME_HCE. CV potential range, 1.8–2.8 V vs Li^+^/Li; scan rate, 0.1 mV s^–1^. EIS carried out at the OCV of the cells and after 1, 5, and 10 voltammetry cycles (inset reports magnification); frequency range, 500 kHz–100 mHz; alternate voltage signal amplitude, 10 mV.

The DEGDME_HCE and TREGDME_HCE are initially exploited in lithium cell using the S:Sn 80:20 cathode by means of CV coupled with EIS as reported in Figure 1. The corresponding voltammograms (Figure 1a, c) show the typical profiles expected for the reversible multiple-step Li/S electrochemical process consisting of a first cycle with a different shape compared to the subsequent ones, which are well overlapped into various peaks centered at about 2.4 and below 2.0 V vs Li^+^/Li during cathodic scan and merged between 2.3 and 2.5 V vs Li^+^/Li during the anodic one.^23^ The above peaks correspond to the reduction of sulfur with formation of soluble polysulfides (Li_2_S_*x*_ with *x* ≥ 6 at 2.4 V, and Li_2_S_*x*_ with 6 > *x* > 2 below 2.0 V) during discharge, and to the oxidation back to sulfur during the charge process.^24^ Furthermore, the difference between the first and subsequent cycles is well justified by the EIS performed at the OCV and after 1, 5, and 10 voltammetry cycles for the cells using DEGDME_HCE (Figure 1b) and TREGDME_HCE (Figure 1d). The corresponding Nyquist plots suggest the activation process typical of Li/S cells using a suitable electrolyte associated with the consolidation of a favorable electrode/electrolyte interphase by the ongoing electrochemical process.^26^ This Li/S activation process has been attributed in a previous paper to microstructural modifications of the electrode that allow an enhanced electric contact between sulfur and the conductive carbon support, and lead to an improved conductivity of the electrode/electrolyte interphase.^30^ Indeed, the EIS data evidence a remarkable decrease of the cell impedance from values between 100 and 200 Ω at the OCV (see insets of Figure 1b and d) to values of the order of 10 Ω for DEGDME_HCE (Figure 1b) and 20 Ω for TREGDME_HCE (Figure 1d). An exhaustive summary of the results of the NLLS analyses performed on the Nyquist plots of Figure 1b and Figure 1d is reported in Table 1.^28,29^ Notably, both CV and EIS results indicate differences between the Li/S cells using DEGDME_HCE and TREGDME_HCE; the former shows smoother less-polarized peaks and a slightly lower steady-state impedance with respect to the latter (compare Figure 1a and the inset of Figure 1b with Figure 1c and the inset of Figure 1d, respectively). These differences may be likely associated with favorable effects on the Li/S electrochemical process promoted by the lower solvent viscosity (0.94 g mL^–1^)^31^ and higher conductivity (3.3 × 10^–3^ S cm^–1^) of DEGDME_HCE compared to the TREGDME_HCE (0.98 g mL^–1^ and 8.9 × 10^–4^ S cm^–1^, respectively)^31^ at room temperature, as reported in our previous study.^20^

**Table 1:** NLLS Analyses Performed on the Nyquist Plots Reported in Figure 1b and d Recorded upon CV Measurements of Li/Electrolyte/S:Sn 80:20 Cells Employing Either DEGDME_HCE (Figure 1b) or TREGDME_HCE (Figure 1d)^28,29^

electrolyte	cell condition	circuit	*R*_1_ [Ω]	*R*_2_ [Ω]	*R*_1_ + *R*_2_ [Ω]	χ^2^
DEGDME_HCE	OCV	R_e_(R_1_Q_1_)(R_2_Q_2_)	103 ± 2	129 ± 3	232 ± 4	1 × 10^–4^
	1 cycle	R_e_(R_1_Q_1_)(R_2_Q_2_)Q_3_	9.9 ± 0.1	3.4 ± 0.2	13.3 ± 0.2	2 × 10^–5^
	5 cycles	R_e_(R_1_Q_1_)(R_2_Q_2_)Q_3_	10.1 ± 0.1	0.8 ± 0.1	10.9 ± 0.1	3 × 10^–5^
	10 cycles	R_e_(R_1_Q_1_)(R_2_Q_2_)Q_3_	9.8 ± 0.5	1.5 ± 0.5	11.3 ± 0.7	6 × 10^–5^
TREGDME_HCE	OCV	R_e_(R_1_Q_1_)(R_2_Q_2_)Q_3_	73.3 ± 0.4	27 ± 3	101 ± 3	5 × 10^–5^
	1 cycle	R_e_(R_1_Q_1_)(R_2_Q_2_)Q_3_	13.1 ± 0.3	9.0 ± 1.5	22.1 ± 1.5	4 × 10^–5^
	5 cycles	R_e_(R_1_Q_1_)(R_2_Q_2_)Q_3_	5.3 ± 0.1	7.2 ± 0.3	12.5 ± 0.3	2 × 10^–5^
	10 cycles	R_e_(R_1_Q_1_)(R_2_Q_2_)Q_3_	5.4 ± 0.1	6.8 ± 0.3	12.2 ± 0.3	2 × 10^–5^

**Figure 2:**
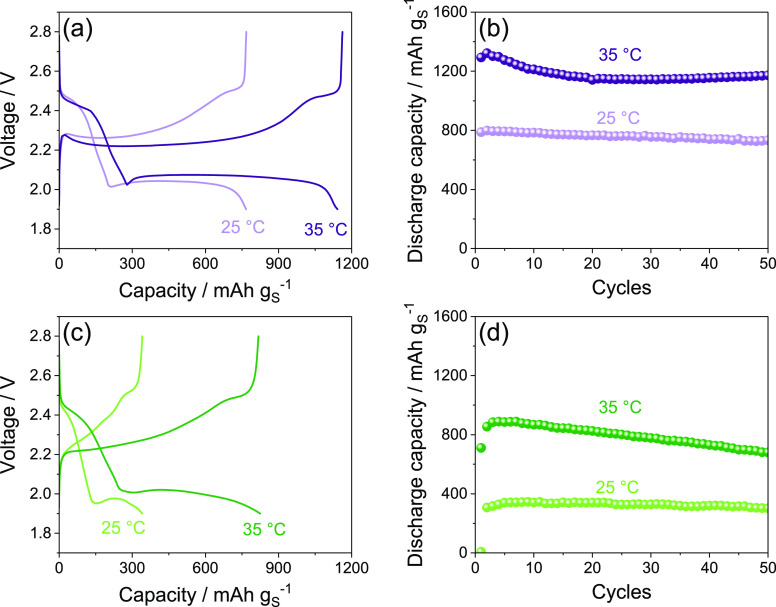
(a, c) Selected voltage profiles and (b, d) corresponding cycling trends at 25 and 35 °C of Li/electrolyte/S:Sn 80:20 cells employing either (a, b) DEGDME_HCE or (c, d) TREGDME_HCE. The cells are galvanostatically cycled using a voltage range between 1.9 and 2.8 V at the constant current rate of C/5 (1C = 1675 mA g_S_^–1^).

Figure 2 displays the performance of DEGDME_HCE (Figure 2a, b) and TREGDME_HCE (Figure 2c, d) in a Li/S:Sn 80:20 cell, cycled at the constant rate of C/5 (1C = 1675 mA g_S_^–1^) at 25 and 35 °C. The selected voltage profiles related to the steady state of the cells (Figure 2a, c) reveal the characteristic response of a Li/S battery, in agreement with CVs of Figure 1, where the two distinct discharge plateaus around 2.4 and 2.0 V ascribed to the formation of long chain lithium polysulfides (Li_2_S_*x*_ with *x* ≥ 6) and short chain ones (Li_2_S_*x*_ with 6 > *x* > 2), respectively, are reversed into two charge plateaus above 2.3 V.^30,32^ Furthermore, the figure shows for both DEGDME_HCE (Figure 2a) and TREGDME_HCE (Figure 2c) a relatively high polarization at room temperature (25 °C), in particular for the latter electrolyte, leading to steady-state specific capacities of about 800 mAh g_S_^–1^ and 340 mAh g_S_^–1^, respectively. The poor response at room temperature of the Li/S cells is most likely due to the hindering of the insulating sulfur kinetics by the high viscosity of the concentrated electrolytes, which is particularly relevant in the case of the TREGDME_HCE due to its longer ether chain, higher lithium salts concentration (see Experimental Section), and consequently higher viscosity compared to DEGDME_HCE.^20,33^ In order to favor the electrochemical kinetics and achieve better performances, subsequent galvanostatic cycling tests were performed on the Li/S:Sn 80:20 cells at a higher temperature, that is, 35 °C, by employing the same C-rate of C/5. Advantageously, the increase of temperature leads to higher capacity values and to lower polarization for both DEGDME_HCE (Figure 2a) and TREGDME_HCE (Figure 2c), as expected by the decrease of the electrolytes viscosity and the concomitant rise of their Li^+^ ions conductivity.^20^

In particular, the cycling trends reported in Figure 2b and d reveal that the cell using DEGDME_HCE delivers at 35 °C a maximum specific capacity of about 1320 mAh g_S_^–1^ (Figure 2b), while the one exploiting TREGDME_HCE exhibits a value approaching 890 mAh g_S_^–1^ (Figure 2d). Furthermore, the cell using DEGDME_HCE shows over the 50 cycles of the tests an excellent retention of the maximum capacity with values ranging from 92% at room temperature to 90% at 35 °C (Figure 2b), while lower but still satisfactory values of 88% at room temperature and 77% at 35 °C are observed for the cell using the more-viscous TREGDME_HCE (Figure 2d).

**Figure 3:**
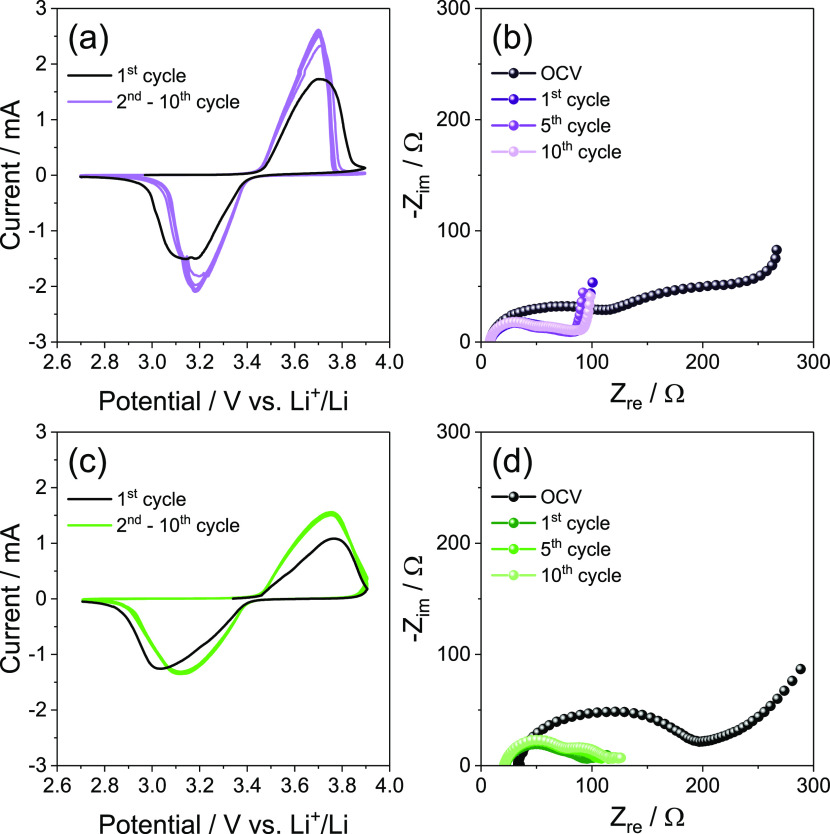
(a, c) Cyclic voltammetry (CV) and (b, d) electrochemical impedance spectroscopy (EIS) measurements performed on Li/electrolyte/LFP cells employing either (a, b) DEGDME_HCE or (c, d) TREGDME_HCE. CV potential range, 2.7–3.9 V vs Li^+^/Li; scan rate, 0.1 mV s^–1^. EIS carried out at the OCV of the cells and after 1, 5, and 10 voltammetry cycles; frequency range, 500 kHz–100 mHz; alternate voltage signal amplitude, 10 mV.

A further application of the electrolytes is exploited by lithium–metal cells using an olivine-structured, (de)insertion LiFePO_4_ (LFP) cathode^3^ which is identified by literature works as a promising candidate for lithium batteries using concentrated solutions.^34,35^ A combined study using CV and EIS is therefore performed and reported in Figure 3, analogously to the investigation provided for the sulfur-based electrode (compare with Figure 1). The voltammograms of the cells using DEGDME_HCE and TREGDME_HCE (Figure 3a and c, respectively) show the characteristic profile centered at about 3.45 V vs Li^+^/Li associated with the deinsertion of Li from the LiFePO_4_ during the anodic scan and its insertion back into the olivine structure during the cathodic scan.^3,25^ The first CV cycle shows a charge/discharge polarization of about 0.3 V vs Li^+^/Li for DEGDME_HCE (Figure 3a, black curve) and of about 0.4 V vs Li^+^/Li for TREGDME_HCE (Figure 3c, black curve). This relatively high polarization may be ascribed to a not yet completely optimized electrode/electrolyte interphase between the LFP electrode and the highly concentrated electrolytes.^10^ Furthermore, the subsequent cycles reveal for the two electrolytes a certain improvement with favorable decrease of the above-mentioned polarization, leading to a shift of about 0.1 V vs Li^+^/Li of the cathodic and anodic peaks. This enhancement is likely justified by the EIS Nyquist plots reported in Figure 3b for DEGDME_HCE and in Figure 3d for TREGDME_HCE and by the results of the corresponding NLLS analyses listed in Table 2.^28,29^ Indeed, the data indicate for the two electrolytes a series of semicircles and lines ascribed to SEI layers, charge transfer processes, and diffusion phenomena occurring in the lithium cells at the various frequencies, with an overall initial resistance of about 300 Ω for DEGDME_HCE and 150 Ω for TREGDME_HCE decreasing to about 90 Ω and 80 Ω, respectively, upon the 10 CV cycles taken under consideration. This likely indicates a partial dissolution or a modification of the pristine SEI layer formed at the electrodes surface by the ongoing nature of the cycling, which can initially favor the kinetics of the electrochemical processes.^7,36^

**Table 2:** NLLS Analyses Performed on the Nyquist Plots Reported in Figure 3b and d Recorded upon CV Measurements of Li/Electrolyte/LFP Cells Employing Either DEGDME_HCE (Figure 3b) or TREGDME_HCE (Figure 3d)^28,29^

electrolyte	cell condition	circuit	*R*_1_ [Ω]	*R*_2_ [Ω]	*R*_1_ + *R*_2_ [Ω]	χ^**2**^
DEGDME_HCE	OCV	R_e_(R_1_Q_1_)(R_2_Q_2_)Q_3_	99 ± 7	207 ± 30	306 ± 31	2 × 10^–4^
	1 cycle	R_e_(R_1_Q_1_)(R_2_Q_2_)Q_3_	37 ± 4	45 ± 6	82 ± 7	8 × 10^–5^
	5 cycles	R_e_(R_1_Q_1_)(R_2_Q_2_)Q_3_	30 ± 2	50 ± 3	79 ± 4	7 × 10^–5^
	10 cycles	R_e_(R_1_Q_1_)(R_2_Q_2_)Q_3_	28 ± 3	60 ± 4	88 ± 5	7 × 10^–5^
TREGDME_HCE	OCV	R_e_(R_1_Q_1_)(R_2_Q_2_)Q_3_	103 ± 10	42 ± 9	145 ± 13	1 × 10^–5^
	1 cycle	R_e_(R_1_Q_1_)(R_2_Q_2_)Q_3_	42 ± 1	15 ± 2	56 ± 2	4 × 10^–5^
	5 cycles	R_e_(R_1_Q_1_)(R_2_Q_2_)Q_3_	45 ± 1	25 ± 1	70 ± 1	3 × 10^–5^
	10 cycles	R_e_(R_1_Q_1_)(R_2_Q_2_)Q_3_	47 ± 1	32 ± 1	79 ± 1	2 × 10^–5^

**Figure 4:**
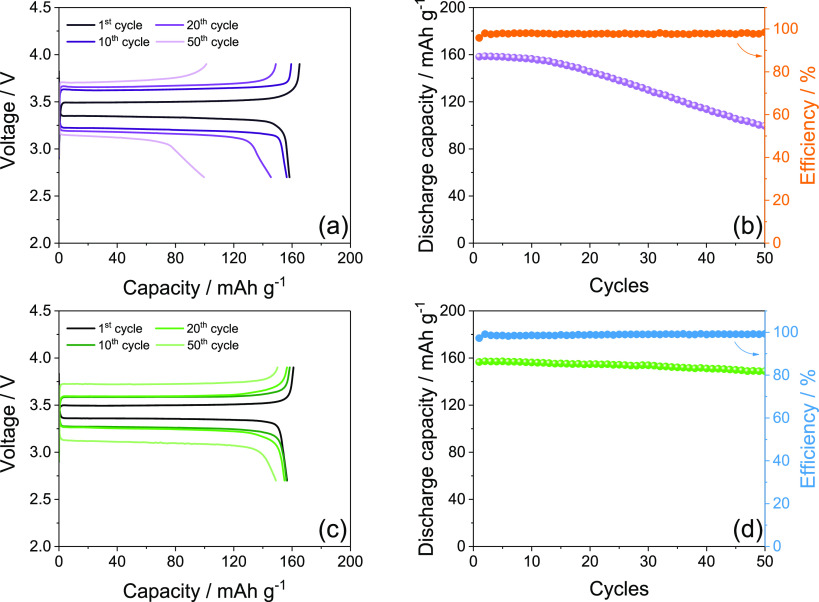
(a, c) Voltage profiles and (b, d) corresponding cycling trends with Coulombic efficiency (right *y*-axis) related to Li/electrolyte/LFP cells employing either (a, b) DEGDME_HCE or (c, d) TREGDME_HCE galvanostatically cycled at the constant current rate of C/5 (1C = 170 mA g^–1^) at room temperature (25 °C). Voltage range, 2.7–3.9 V.

The DEGDME_HCE and TREGDME_HCE are subsequently employed in a Li/LFP cell and galvanostatically cycled at the constant current rate of C/5 (1C = 170 mA g^–1^) at room temperature, with the outcomes displayed in Figure 4. The voltage profiles reported in Figure 4a (DEGDME_HCE) and Figure 4c (TREGDME_HCE) reflect the response associated with the LiFePO_4_ ⇄ Li + FePO_4_ electrochemical process, centered at about 3.45 V as already described in CVs of Figure 3.^27,37^ The cells show relatively limited polarization during the first cycle and capacity values approaching 160 mAh g^–1^, that is, about 94% of the theoretical value. Moreover, the Coulombic efficiency interestingly approaches 100% during both tests, as displayed by the cycling trends in Figure 4b for DEGDME_HCE and in Figure 4d for TREGDME_HCE, while a progressive increase of the polarization affects the cells after 10 cycles and leads to capacity decay upon 50 cycles, which is more remarkable for the former (Figure 4a) compared to the latter electrolyte (Figure 4c). Therefore, the cell using DEGDME_HCE (Figure 4b) exhibits a capacity retention of 63% during the 50 charge–discharge cycles taken into account; instead, the one using TREGDME_HCE (Figure 4d) holds 94% of the initial capacity upon the same number of cycles. This behavior can be ascribed to the nature of the SEI layer formed at the electrode surface upon cycling, which is influenced by the electrolyte composition, by the lithium salts content, and by the operating conditions.^38−40^ In particular, the higher lithium salts concentration of TREGDME_HCE compared to DEGDME_HCE could play a crucial role in the formation of a more suitable SEI in this cell, as already suggested by its application in a Li/O_2_ cell studied elsewhere.^20^ A further reason for the different Li/LFP cell performances between the two solutions may be the narrower electrochemical stability window of DEGDME_HCE (0–4.3 V) with respect to TREGDME_HCE (0–4.4 V).^20^ However, the relevant increase in cell polarization observed using both DEGDME_HCE (Figure 4a) and TREGDME_HCE (Figure 4c) after 50 cycles may actually indicate the need for further optimization of the SEI at the electrodes surface to allow proper operation of the glyme-based electrolytes in a lithium–metal cell using insertion cathodes. Indeed, previous literature has demonstrated that high concentrations of lithium salts in glyme-based electrolytes can lead to an uneven composition and low thickness of the SEI layer, possibly leading to a modest stability.^41^ We have demonstrated in a recent paper that TREGDME dissolving lithium trifluoromethanesulfonate (LiCF_3_SO_3_) and LiNO_3_ in conventional concentrations undergoes an electrochemical optimization process in a Li/LFP cell by adopting a reduction step at the first discharge occurring around 1.5 V, i.e., a voltage value far lower than the ones exploited in the galvanostatic measurements reported in Figure 4.^36^ The above-mentioned reduction deals with LiNO_3_ and actually leads to the formation of stable interfaces at the electrodes surface with a remarkable improvement of the cell performance.^7,36^

**Figure 5:**
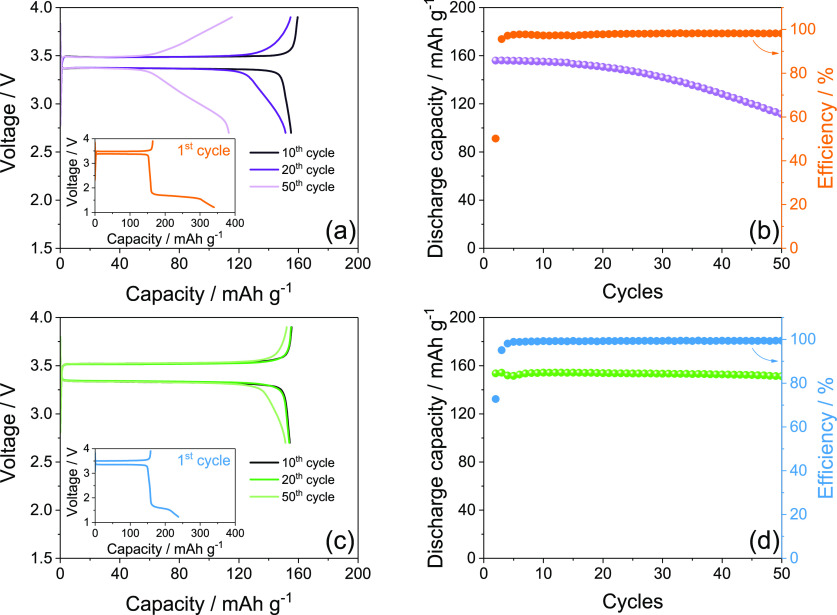
(a, c) Voltage profiles and (b, d) corresponding cycling trends with Coulombic efficiency (right *y*-axis) related to Li/electrolyte/LFP cells employing either (a, b) DEGDME_HCE or (c, d) TREGDME_HCE galvanostatically cycled at room temperature (25 °C) at the constant current rate of C/5 (1C = 170 mA g^–1^) in a voltage range between 1.2 and 3.9 V for the first cycle (inset in panels (a) and (c)) and between 2.7 and 3.9 V for the subsequent ones.

Therefore, an additional galvanostatic test was performed using DEGDME_HCE and TREGDME_HCE in Li/LFP cells at the constant current rate of C/5 by employing a voltage range between 1.2 and 3.9 V for the first cycle and between 2.7 and 3.9 V for the subsequent ones, as reported in Figure 5. Insets in Figure 5a (DEGDME_HCE) and Figure 5c (TREGDME_HCE) show the voltage profile related to the first charge–discharge cycle, and they reveal the evolution of a discharge plateau between 1.5 and 1.7 V due to the reduction of LiNO_3_;^7,36^ the subsequent voltage profiles are reported in Figure 5a and c, respectively. The cycling trends of the above Li/LFP cells evidence initial capacity values approaching 160 mAh g^–1^ and Coulombic efficiency around 100% upon the first cycles for both DEGDME_HCE (Figure 5b) and TREGDME_HCE (Figure 5d). However, the cell employing DEGDME_HCE (Figure 5b) still exhibits a certain capacity decay, despite retention increases from 63% to 71% compared to the analogue test performed without the additional reduction step (compare Figure 4b and Figure 5b) upon the 50 cycles taken into account. Furthermore, the voltage profiles of the cell using DEGDME_HCE (Figure 5a) do not show the increase in cell polarization during cycling observed in the previous test (compare Figure 4a and Figure 5a), while a slope appears at the end of the charge and the discharge profiles after 20 cycles and becomes more relevant after 50 cycles. The decrease of cell capacity and the appearance of the slope at the end of the (de)insertion processes of LiFePO_4_ may be associated with an excessive growth of the SEI layer at the electrodes surface and possibly with gradual changes in cathode crystallite size distribution and surface free energies of the lithiated and delithiated phases, which lead to a change of the biphasic potential.^11^ Instead, the cell employing TREGDME_HCE shows a capacity retention increasing from 94% of the previous test (Figure 4d) up to 97% (Figure 5d), while the corresponding voltage profiles reveal only a slight slope after 50 cycles without any sign of polarization increase or deterioration (Figure 5c). Therefore, we may suggest the additional reduction step at low voltage during the first cycle as an actual strategy to improve the performance of the lithium–metal cell using concentrated glyme-based electrolytes with the LFP electrode, particularly those having a longer ether chain such as TREGDME.

**Figure 6:**
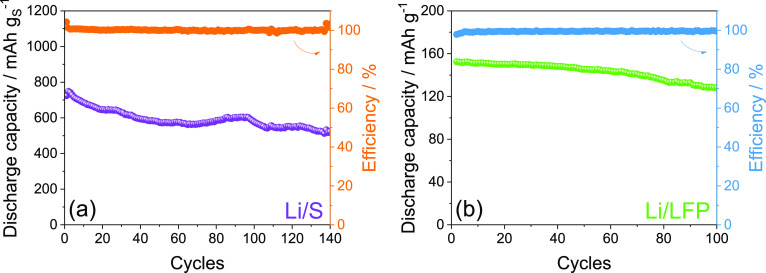
Cycling trends with Coulombic efficiency (right *y*-axis) related to (a) Li/DEGDME_HCE/S:Sn 80:20 and (b) Li/TREGDME_HCE/LFP cells galvanostatically cycled at 1C (1675 mA g_S_^–1^) and C/3 (1C = 170 mA g^–1^), respectively. The Li/S cell was cycled at 35 °C by exploiting an electrolyte/sulfur ratio of 20 μL mg^–1^ and a 1.6–2.8 V voltage range. The Li/LFP cell was cycled at room temperature (25 °C) by employing a 1.2–3.9 V voltage range for the first cycle and voltage limits of 2.7 and 3.9 V for the subsequent ones.

In order to extend the cycle life, additional galvanostatic cycling tests were performed on Li/S and Li/LFP cells by adopting the most suitable operative conditions according to the data reported in this work. Indeed, Figure 6 reports the cycling trends of a Li/DEGDME_HCE/S:Sn 80:20 cell (Figure 6a) operating at 35 °C and of a Li/TREGDME_HCE/LFP cell (Figure 6b) working at room temperature (25 °C), the latter by exploiting the initial reduction step at low voltage (1.2 V). It is worth mentioning that the Li/S cell adopted an electrolyte/sulfur ratio limited to 20 μL mg^–1^ in order to reduce the excess of electrolyte and, thus, to increase the practical energy density of the device.^30^ As observed in Figure 6, both cells exhibit notable performances, long cycle life, and Coulombic efficiency around 100% even by cycling at higher current rates, that is, at 1C for the Li/S cell (1675 mA g_S_^–1^) and C/3 for the Li/LFP one (1C = 170 mA g^–1^). In particular, the Li/S cell delivers 140 cycles with an initial capacity upon activation of almost 750 mAh g_S_^–1^ retained at the 70% at the end of the test (Figure 6a), while the Li/LFP cell displays a capacity of 152 mAh g^–1^ (89% of the theoretical value) retained at the 85% after 100 charge/discharge cycles (Figure 6b). Despite the lower delivered capacity values with respect to the tests performed at C/5 (see Figures 2 and 5), as expected by the employment of higher currens, these tests further evidence that the optimal tuning of the working conditions can lead to the extension of the cycle life and to a notable as well as steady delivered capacity values of Li/S and Li/LFP cells with lowly flammable concentrated glyme-based electrolytes.

## Conclusions

Glyme-based electrolytes with two different chain lengths employing high lithium salts concentrations (indicated as DEGDME_HCE and TREGDME_HCE) are studied in either Li/S or Li/LFP cells to explore the applicability of this class of solutions in a safe and high-performance Li-metal battery. The CV tests performed on the Li/S cells revealed for both electrolytes a reversible electrochemical process centered at about 2.1 and 2.4 V and an activation process leading to the decrease of the impedance from values of the order of 100 Ω to about 10 Ω upon cycling. Furthermore, galvanostatic measurements of the Li/S cells carried out using the constant current rate of C/5 at 25 and 35 °C indicated for DEGDME_HCE capacities of about 800 and 1300 mAh g_S_^–1^, respectively, while lower values of about 340 and 890 mAh g_S_^–1^ were obtained for TREGDME_HCE. The more relevant performances of the Li/S cells using DEGDME_HCE compared to TREGDME_HCE were attributed to faster charge transfer kinetics in the former electrolyte compared to the latter. Meanwhile, the lithium cells employing the LFP electrode suggested the two solutions as possible electrolyte media for the reversible (de)insertion process at about 3.45 V; however, the tests indicated possible issues ascribed to the SEI layer formed at the electrodes surface, leading to a polarization increase by cell cycling. These issues were relevantly mitigated, in particular when using TREGDME_HCE, by adopting a first discharge of the cell extended down to 1.2 V in order to promote a further reduction of the LiNO_3_ additive and the consolidation of a suitable SEI layer. Therefore, the above Li/LFP cells delivered at C/5 rate an initial capacity of about 160 mAh g^–1^ (94% of the theoretical value), an efficiency approaching 100%, and a capacity retention of 71% for DEGDME_HCE and 97% for TREGDME_HCE upon 50 charge/discharge cycles. Furthermore, Li/DEGDME_HCE/S and Li/TREGDME_HCE/LFP cells operating with the most adequate conditions have shown satisfactory performances at current rates increased to 1C and C/3, respectively. The former cell delivered 750 mAh g_S_^–1^ with capacity retention of 70% over 140 cycles at 35 °C, while the latter exhibited about 150 mAh g^–1^ with a retention of 85% after 100 cycles at 25 °C by exploiting the initial reduction step at 1.2 V.

In summary, the findings of this work suggested the possible use of concentrated solutions based on end-capped glymes in efficient lithium–metal cells by careful tuning of (i) the ether chain length, (ii) the salt nature and concentration, (iii) the chemistry of the cathode material, and (iv) the operative conditions, including temperature and voltage limits. In addition, the intrinsically lower flammability of the concentrated glymes reported herein compared to the common electrolytes used in cell is expected to allow the development of a lithium–metal battery with high energy and acceptable safety content.

## References

[ref1] Abraham K. M. M. Prospects and Limits of Energy Storage in Batteries. J. Phys. Chem. Lett. 2015, 6 (5), 830–844. 10.1021/jz5026273. 26262660

[ref2] Lu L.; Han X.; Li J.; Hua J.; Ouyang M. A Review on the Key Issues for Lithium-Ion Battery Management in Electric Vehicles. J. Power Sources 2013, 226, 272–288. 10.1016/j.jpowsour.2012.10.060.

[ref3] Di Lecce D.; Verrelli R.; Hassoun J. Lithium-Ion Batteries for Sustainable Energy Storage: Recent Advances towards New Cell Configurations. Green Chem. 2017, 19 (15), 3442–3467. 10.1039/C7GC01328K.

[ref4] Scrosati B.; Garche J. Lithium Batteries: Status, Prospects and Future. J. Power Sources 2010, 195 (9), 2419–2430. 10.1016/j.jpowsour.2009.11.048.

[ref5] Varzi A.; Thanner K.; Scipioni R.; Di Lecce D.; Hassoun J.; Dörfler S.; Altheus H.; Kaskel S.; Prehal C.; Freunberger S. A. Current Status and Future Perspectives of Lithium Metal Batteries. J. Power Sources 2020, 480, 228803 10.1016/j.jpowsour.2020.228803.

[ref6] Goodenough J. B.; Kim Y. Challenges for Rechargeable Li Batteries. Chem. Mater. 2010, 22 (3), 587–603. 10.1021/cm901452z.

[ref7] Carbone L.; Gobet M.; Peng J.; Devany M.; Scrosati B.; Greenbaum S.; Hassoun J. Polyethylene Glycol Dimethyl Ether (PEGDME)-Based Electrolyte for Lithium Metal Battery. J. Power Sources 2015, 299, 460–464. 10.1016/j.jpowsour.2015.08.090.

[ref8] Zhang S. S. Role of LiNO_3_ in Rechargeable Lithium/Sulfur Battery. Electrochim. Acta 2012, 70, 344–348. 10.1016/j.electacta.2012.03.081.

[ref9] Jozwiuk A.; Berkes B. B.; Weiß T.; Sommer H.; Janek J.; Brezesinski T. The Critical Role of Lithium Nitrate in the Gas Evolution of Lithium–sulfur Batteries. Energy Environ. Sci. 2016, 9 (8), 2603–2608. 10.1039/C6EE00789A.

[ref10] Derrien G.; Hassoun J.; Sacchetti S.; Panero S. Nanocomposite PEO-Based Polymer Electrolyte Using a Highly Porous, Super Acid Zirconia Filler. Solid State Ionics 2009, 180, 1267–1271. 10.1016/j.ssi.2009.07.006.

[ref11] Wei S.; Inoue S.; Di Lecce D.; Li Z.; Tominaga Y.; Hassoun J. Towards a High-Performance Lithium-Metal Battery with Glyme Solution and an Olivine Cathode. ChemElectroChem 2020, 7 (11), 2344–2344. 10.1002/celc.202000554.

[ref12] Carbone L.; Gobet M.; Peng J.; Devany M.; Scrosati B.; Greenbaum S.; Hassoun J. Comparative Study of Ether-Based Electrolytes for Application in Lithium-Sulfur Battery. ACS Appl. Mater. Interfaces 2015, 7 (25), 13859–13865. 10.1021/acsami.5b02160. 26057152

[ref13] Tobishima S.; Morimoto H.; Aoki M.; Saito Y.; Inose T.; Fukumoto T.; Kuryu T. Glyme-Based Nonaqueous Electrolytes for Rechargeable Lithium Cells. Electrochim. Acta 2004, 49 (6), 979–987. 10.1016/j.electacta.2003.10.009.

[ref14] Liu X.; Zarrabeitia M.; Qin B.; Elia G. A.; Passerini S. Cathode–Electrolyte Interphase in a LiTFSI/Tetraglyme Electrolyte Promoting the Cyclability of V_2_O_5_. ACS Appl. Mater. Interfaces 2020, 12 (49), 54782–54790. 10.1021/acsami.0c16727. 33216545 PMC9159652

[ref15] Benítez A.; Marangon V.; Hernández-Rentero C.; Caballero Á.; Morales J.; Hassoun J. Porous Cr_2_O_3_@C Composite Derived from Metal Organic Framework in Efficient Semi-Liquid Lithium-Sulfur Battery. Mater. Chem. Phys. 2020, 255, 123484 10.1016/j.matchemphys.2020.123484.

[ref16] Xie J.-D.; Liu W.-J.; Li C.; Patra J.; Gandomi Y. A.; Dong Q.-F.; Chang J.-K. Superior Coulombic Efficiency of Lithium Anodes for Rechargeable Batteries Utilizing High-Concentration Ether Electrolytes. Electrochim. Acta 2019, 319, 625–633. 10.1016/j.electacta.2019.07.020.

[ref17] Adams B. D.; Carino E. V.; Connell J. G.; Han K. S.; Cao R.; Chen J.; Zheng J.; Li Q.; Mueller K. T.; Henderson W. A.; Zhang J.-G. Long Term Stability of Li-S Batteries Using High Concentration Lithium Nitrate Electrolytes. Nano Energy 2017, 40, 607–617. 10.1016/j.nanoen.2017.09.015.

[ref18] Ren X.; Zou L.; Cao X.; Engelhard M. H.; Liu W.; Burton S. D.; Lee H.; Niu C.; Matthews B. E.; Zhu Z.; Wang C.; Arey B. W.; Xiao J.; Liu J.; Zhang J.-G.; Xu W. Enabling High-Voltage Lithium-Metal Batteries under Practical Conditions. Joule 2019, 3 (7), 1662–1676. 10.1016/j.joule.2019.05.006.

[ref19] Yamada Y.; Yamada A. Review—Superconcentrated Electrolytes for Lithium Batteries. J. Electrochem. Soc. 2015, 162 (14), A2406–A2423. 10.1149/2.0041514jes.

[ref20] Marangon V.; Hernandez-Rentero C.; Levchenko S.; Bianchini G.; Spagnolo D.; Caballero A.; Morales J.; Hassoun J. Lithium–Oxygen Battery Exploiting Highly Concentrated Glyme-Based Electrolytes. ACS Appl. Energy Mater. 2020, 3 (12), 12263–12275. 10.1021/acsaem.0c02331.

[ref21] Aurbach D. Review of Selected Electrode–solution Interactions Which Determine the Performance of Li and Li Ion Batteries. J. Power Sources 2000, 89 (2), 206–218. 10.1016/S0378-7753(00)00431-6.

[ref22] Liu Q.; Cresce A.; Schroeder M.; Xu K.; Mu D.; Wu B.; Shi L.; Wu F. Insight on Lithium Metal Anode Interphasial Chemistry: Reduction Mechanism of Cyclic Ether Solvent and SEI Film Formation. Energy Storage Mater. 2019, 17, 366–373. 10.1016/j.ensm.2018.09.024.

[ref23] Waluś S.; Barchasz C.; Bouchet R.; Leprêtre J.-C.; Colin J.-F.; Martin J.-F.; Elkaïm E.; Baehtz C.; Alloin F. Lithium/Sulfur Batteries Upon Cycling: Structural Modifications and Species Quantification by In Situ and Operando X-Ray Diffraction Spectroscopy. Adv. Energy Mater. 2015, 5 (16), 1500165 10.1002/aenm.201500165.

[ref24] Di Lecce D.; Marangon V.; Du W.; Brett D. J. L.; Shearing P. R.; Hassoun J. The Role of Synthesis Pathway on the Microstructural Characteristics of Sulfur-Carbon Composites: X-Ray Imaging and Electrochemistry in Lithium Battery. J. Power Sources 2020, 472, 228424 10.1016/j.jpowsour.2020.228424.

[ref25] Hernández-Rentero C.; Marangon V.; Olivares-Marín M.; Gómez-Serrano V.; Caballero Á.; Morales J.; Hassoun J. Alternative Lithium-Ion Battery Using Biomass-Derived Carbons as Environmentally Sustainable Anode. J. Colloid Interface Sci. 2020, 573, 396–408. 10.1016/j.jcis.2020.03.092. 32304949

[ref26] Marangon V.; Hassoun J. Sulfur Loaded by Nanometric Tin as a New Electrode for High-Performance Lithium/Sulfur Batteries. Energy Technol. 2019, 7 (12), 1900081 10.1002/ente.201900081.

[ref27] Brutti S.; Hassoun J.; Scrosati B.; Lin C.-Y.; Wu H.; Hsieh H.-W. A High Power Sn–C/C–LiFePO_4_ Lithium Ion Battery. J. Power Sources 2012, 217, 72–76. 10.1016/j.jpowsour.2012.05.102.

[ref28] Boukamp B. A Nonlinear Least Squares Fit Procedure for Analysis of Immittance Data of Electrochemical Systems. Solid State Ionics 1986, 20 (1), 31–44. 10.1016/0167-2738(86)90031-7.

[ref29] Boukamp B. A. A Package for Impedance/Admittance Data Analysis. Solid State Ionics 1986, 18–19, 136–140. 10.1016/0167-2738(86)90100-1.

[ref30] Marangon V.; Di Lecce D.; Orsatti F.; Brett D. J. L.; Shearing P. R.; Hassoun J. Investigating High-Performance Sulfur–metal Nanocomposites for Lithium Batteries. Sustain. Energy Fuels 2020, 4 (6), 2907–2923. 10.1039/D0SE00134A.

[ref31] Riadigos C. F.; Iglesias R.; Rivas M. A.; Iglesias T. P. Permittivity and Density of the Systems (Monoglyme, Diglyme, Triglyme, or Tetraglyme+n-Heptane) at Several Temperatures. J. Chem. Thermodyn. 2011, 43 (3), 275–283. 10.1016/j.jct.2010.09.008.

[ref32] Barchasz C.; Molton F.; Duboc C.; Leprêtre J.-C.; Patoux S.; Alloin F. Lithium/Sulfur Cell Discharge Mechanism: An Original Approach for Intermediate Species Identification. Anal. Chem. 2012, 84 (9), 3973–3980. 10.1021/ac2032244. 22482872

[ref33] Yoshida K.; Tsuchiya M.; Tachikawa N.; Dokko K.; Watanabe M. Change from Glyme Solutions to Quasi-Ionic Liquids for Binary Mixtures Consisting of Lithium Bis(Trifluoromethanesulfonyl)Amide and Glymes. J. Phys. Chem. C 2011, 115 (37), 18384–18394. 10.1021/jp206881t.

[ref34] Liu X.; Shen C.; Gao N.; Hou Q.; Song F.; Tian X.; He Y.; Huang J.; Fang Z.; Xie K. Concentrated Electrolytes Based on Dual Salts of LiFSI and LiODFB for Lithium-Metal Battery. Electrochim. Acta 2018, 289, 422–427. 10.1016/j.electacta.2018.09.085.

[ref35] Liu P.; Ma Q.; Fang Z.; Ma J.; Hu Y.-S.; Zhou Z.-B.; Li H.; Huang X.-J.; Chen L.-Q. Concentrated Dual-Salt Electrolytes for Improving the Cycling Stability of Lithium Metal Anodes. Chin. Phys. B 2016, 25 (7), 078203 10.1088/1674-1056/25/7/078203.

[ref36] Carbone L.; Di Lecce D.; Gobet M.; Munoz S.; Devany M.; Greenbaum S.; Hassoun J. Relevant Features of a Triethylene Glycol Dimethyl Ether-Based Electrolyte for Application in Lithium Battery. ACS Appl. Mater. Interfaces 2017, 9 (20), 17085–17095. 10.1021/acsami.7b03235. 28440629

[ref37] Marangon V.; Tominaga Y.; Hassoun J. An Alternative Composite Polymer Electrolyte for High Performances Lithium Battery. J. Power Sources 2020, 449, 227508 10.1016/j.jpowsour.2019.227508.

[ref38] Camacho-Forero L. E.; Smith T. W.; Balbuena P. B. Effects of High and Low Salt Concentration in Electrolytes at Lithium–Metal Anode Surfaces. J. Phys. Chem. C 2017, 121 (1), 182–194. 10.1021/acs.jpcc.6b10774.

[ref39] Yamada Y. Developing New Functionalities of Superconcentrated Electrolytes for Lithium-Ion Batteries. Electrochemistry 2017, 85 (9), 559–565. 10.5796/electrochemistry.85.559.

[ref40] Winter M. The Solid Electrolyte Interphase – The Most Important and the Least Understood Solid Electrolyte in Rechargeable Li Batteries. Z. Phys. Chem. 2009, 223 (10–11), 1395–1406. 10.1524/zpch.2009.6086.

[ref41] Ruggeri I.; La Monaca A.; De Giorgio F.; Soavi F.; Arbizzani C.; Berbenni V.; Ferrara C.; Mustarelli P. Correlating Structure and Properties of Super-Concentrated Electrolyte Solutions: ^17^O NMR and Electrochemical Characterization. ChemElectroChem 2019, 6 (15), 4002–4009. 10.1002/celc.201900829.

